# Therapeutic Response of CCKBR-Positive Tumors to Combinatory Treatment with Everolimus and the Radiolabeled Minigastrin Analogue [^177^Lu]Lu-PP-F11N

**DOI:** 10.3390/pharmaceutics13122156

**Published:** 2021-12-15

**Authors:** Michal Grzmil, Stefan Imobersteg, Alain Blanc, Stephan Frank, Roger Schibli, Martin P. Béhé

**Affiliations:** 1 Center for Radiopharmaceutical Sciences ETH-PSI-USZ, Paul Scherrer Institute, 5232 Villigen, Switzerland; stefan.imobersteg@psi.ch (S.I.); alain.blanc@psi.ch (A.B.); roger.schibli@psi.ch (R.S.); martin.behe@psi.ch (M.P.B.); 2 Division of Neuropathology, Institute of Pathology, University of Basel, 4031 Basel, Switzerland; stephan.frank@usb.ch; 3 Department of Chemistry and Applied Biosciences, ETH Zurich, 8093 Zurich, Switzerland

**Keywords:** cholecystokinin B receptor (CCKBR), CCK2R, minigastrin analogue PP-F11N, everolimus, RAD001, mammalian target of rapamycin complex 1 (mTORC1)

## Abstract

The inhibition of the mammalian target of rapamycin complex 1 (mTORC1) by everolimus (RAD001) was recently shown to enhance the tumor uptake of radiolabeled minigastrin. In this paper, we investigate if this finding can improve the in vivo therapeutic response to [^177^Lu]Lu-PP-F11N treatment. The N-terminal DOTA-conjugated gastrin analogue PP-F11N (DOTA-(DGlu)_6_-Ala-Tyr-Gly-Trp-Nle-Asp-Phe) was used to evaluate treatment efficacy in the human A431/CCKBR xenograft nude mouse model in combination with RAD001. Both RAD001 and [^177^Lu]Lu-PP-F11N single treatments as well as their combination inhibited tumor growth and increased survival. In concomitantly treated mice, the average tumor size and median survival time were significantly reduced and extended, respectively, as compared to the monotherapies. The histological analysis of kidney and stomach dissected after treatment with RAD001 and [^177^Lu]Lu-PP-F11N did not indicate significant adverse effects. In conclusion, our study data demonstrate the potential of mTORC1 inhibition to substantially improve the therapeutic efficacy of radiolabeled minigastrin analogues in CCKBR-positive cancers.

## 1. Introduction

Radiolabeled minigastrin analogues bind with high affinity to the cholecystokinin B receptor (CCKBR, also known as CCK2R), whose overexpression was previously demonstrated by a radioligand binding assay in human cancer tissue sections, including medullary thyroid cancer (MTC), small cell lung cancer, astrocytoma, and stromal ovarian cancer [1]. CCKBR belongs to the family of G-protein-coupled receptors (GPCRs) and its activation was further reported in pancreatic and colorectal cancer, in which CCKBR signaling played an important role in cancer cell proliferation [2,3].

The favorable pharmacokinetics of radiolabeled minigastrin analogues were verified in human xenograft rodent models and showed PP-F11N or CP04 to be promising radiopharmaceuticals for theranostic applications in CCKBR-positive neuroendocrine tumors [4,5]. The first clinical application of lutetium-177 labeled minigastrin [^177^Lu]Lu-PP-F11N revealed efficient intratumor accumulation by single photon emission computed tomography (SPECT) in MTC patients [6]. More recently, high tumor accumulation of metabolically stable radiolabeled minigastrin [^177^Lu]Lu-PP-F11N was demonstrated in MTC patients with low adverse reactions [7]. Apart from a low kidney and bone marrow radiation dose, the latter study reported [^177^Lu]Lu-PP-F11N uptake by stomach tissue, which expresses CCKBR, and suggested stomach as a dose-limiting organ with a tumor-to-stomach dose ratio of 3.34. In order to improve the tumor-specific uptake of [^177^Lu]Lu -PP-F11N, in a recent preclinical study, a kinase inhibitor library screen identified a clinically feasible way for augmented tumor uptake by the pharmacological inhibition of the mammalian target of rapamycin complex 1 (mTORC1), which enhanced the CCKBR protein level [8]. In this report, we validate the therapeutic response to a concomitant treatment with mTORC1 inhibitor RAD001 (everolimus) and [^177^Lu]Lu-PP-F11N. Our study data demonstrate enhanced efficacy of radiolabeled minigastrin analogues in RAD001-treated CCKBR-tumor bearing nude mice and suggest further development of the combinatory treatment for clinical applications.

## 2. Materials and Methods

### 2.1. Radiolabeling and Purification of Radiolabeled Minigastrin

The N-terminal DOTA-conjugated gastrin analogue PP-F11N (DOTA-(DGlu)_6_-Ala-Tyr-Gly-Trp-Nle-Asp-Phe) was obtained from PSL GmbH (Heidelberg, Germany), whereas the [^177^Lu]LuCl_3_ solution was obtained from ITG GmbH, Munich, Germany. The labeling reaction (260 µL) contained 2 GBq [^177^Lu]Lu (2.77 nmol) per 83 nmol PP-F11N in a 0.4 M ammonium acetate buffer (pH 5.5). Labeling was carried out at 90 °C for 15 min and 2 µL 0.5 mM EDTA was added to chelate residual radionuclides. The [^177^Lu]Lu incorporation was analyzed by high-performance liquid chromatography (HPLC) using a C18 column, and reached above 99% efficiency (Supplementary Figure S1). Prior to the animal study, the purification of [^177^Lu]Lu-PP-F11N was accomplished by using a Merck Hitachi LaChrom 2D HPLC system as previously described [9], yielding a specific activity of 722 MBq/nmol. The eluted radioactive fractions were concentrated on the SpeedVac and the PBS-diluted radiopeptide was used directly for in vivo experiments.

### 2.2. Tissue Culture and Animal Study

Human squamous carcinoma A431 cells overexpressing human CCKBR (generously provided by Dr. Luigi Aloj) were generated as previously described [10]. Cells were grown in Dulbecco’s Modified Eagle Medium (DMEM) that contained 10% fetal calf serum (FCS), 2 mM glutamine, and antibiotics (0.1 mg/mL streptomycin, 100 IU/mL penicillin) (Bio Concept Ltd., Allschwil, Switzerland) in a humidified incubator at 37 °C and 5% CO_2_. For the in vivo study, 5 × 10^6^ of A431/CCKBR cells were suspended in 100 µL of sterile phosphate-buffered saline (PBS) and injected subcutaneously into isoflurane/oxygen anesthetized CD-1 female nude mice (Charles Rivers, Sulzfeld, Germany). The tumor growth was monitored non-invasively using a caliper. Six days after implantation the average tumor volume reached 0.12 ± 0.04 cm^3^, and the mice were randomly allocated into experimental groups and subjected to the treatments. Everolimus (RAD001) was obtained from Lucerna Chem AG (Luzern, Switzeralnd) and 3 mg/kg was administered via intraperitoneal (IP) injection daily for 5 or 10 days, as indicated. Control mice received PBS via IP injection. The RAD001 dose was based on the previous studies in nude mice, which show an increased uptake of radiolabeled minigastrin as well as anti-tumor activity without adverse effects [8]. HPLC-purified 60 MBq of [^177^Lu]Lu-PPF11N (722 MBq/nmol) in 100 µL PBS was injected intravenously, whereas the control group was injected with 100 µL PBS. Tumor diameter and animal weight were recorded daily. Tumor volumes were calculated using the formula V = (W^2^ × L)/2. Mice were sacrificed when tumor volume exceeded 1.5 cm^3^. Mice with ulcerated tumors, which occurred in all groups, were sacrificed prematurely and excluded from analysis. All experiments were performed in accordance with the Swiss Animal Protection Laws (License no. 75699). GraphPad Prism 7.00 (GraphPad Software, San Diego, CA, USA) for Windows was used for all statistical analyses.

### 2.3. Histopathology

Post-mortem dissected stomachs and kidneys were fixed in formalin and used for preparation of paraffin sections. Tissue slides were subjected to deparaffinization and rehydration followed by a routine Hematoxylin Eosin (H&E) stain as previously reported [8]. Prior to histological assessment, the images were acquired by a slide scanner (Nikon Instruments Europe, Amstelveen, The Netherlands).

### 2.4. Statistics

A one-way ANOVA test combined with two-stage linear step-up procedure of Benjamini, Krieger, and Yekutieli for multiple comparison tests was performed for all treated groups. The log-rank test (also referred as the Cox–Mantel test) was used to analyze survival curves obtained from the treatment groups in comparison with the control group. Humane euthanasia was defined as the endpoint in the survival curves. *p*-value of 0.05 or lower was considered to be statistically significant.

## 3. Results

### 3.1. RAD001 Increases Therapeutic Response to [^177^Lu]Lu-PP-F11N in CCKBR-Tumor Bearing Nude Mice

**Figure 1:**
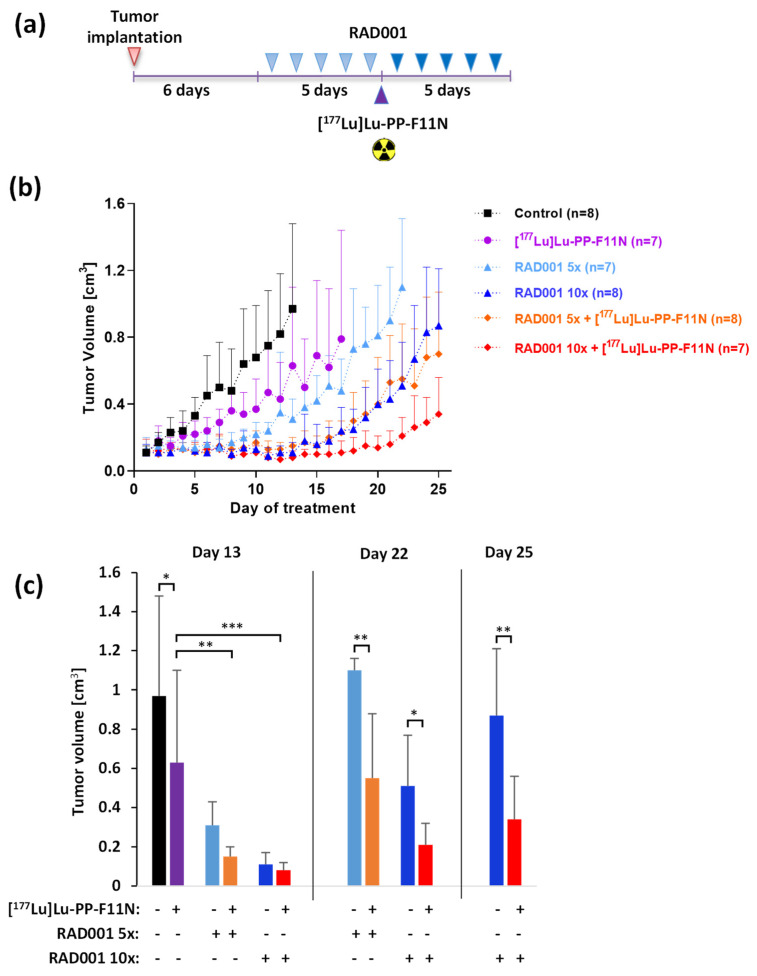
Tumor growth inhibition in RAD001 and [^177^Lu]Lu-PP-F11N-treated mice. (**a**) Experimental design: after the implantation of A431/CCKBR cells into nude mice, 5 or 10 doses of RAD001 were administrated alone or in combination with 60 kBq [^177^Lu]Lu-PP-F11N, as indicated. (**b**) Tumor growth curves of control and treated groups. Data represent mean ± SD. (**c**) Bars represent average tumor volumes ± SD on day 13, 22, and 25 after treatment initiation. * *p* < 0.05, ** *p* < 0.01, *** *p* < 0.001.

To evaluate the therapeutic effects of combinatory treatment, tumor growth and mean survival of immunocompromised A431/CCKBR-tumor bearing nude mice were investigated after the administration of 5 or 10 doses of RAD001 alone or in combination with 60 MBq of [^177^Lu]Lu-PP-F11N, as indicated in (Figure 1a). Both the RAD001 and [^177^Lu]Lu-PP-F11N single treatment as well as their combination significantly inhibited tumor growth (Figure 1b). On day 13, when all mice were still alive in all groups, the average tumor volume in the control group reached 0.97 cm^3^. In contrast, the average tumor sizes in mice treated with [^177^Lu]Lu-PP-F11N, 5 or 10 doses of RAD001, and 5 or 10 doses of RAD001 in combination with [^177^Lu]Lu-PP-F11N were significantly reduced (*p* < 0.05), reaching 0.63, 0.31, or 0.11 and 0.15 or 0.08 cm^3^, respectively (Figure 1c). Tumor growth in 5× or 10× RAD001-treated mice in combination with [^177^Lu]Lu-PP-F11N was significantly reduced, compared to the [^177^Lu]Lu-PP-F11N monotherapy. On day 22, the average tumor size in mice concomitantly treated with 5 or 10 doses of RAD001 and [^177^Lu]Lu-PP-F11N was significantly reduced as compared to mice that received 5 or 10 doses of RAD001 only. On day 25, the average tumor size in mice concomitantly treated with 10 doses of RAD001 and [^177^Lu]Lu-PP-F11N was significantly reduced as compared to the 10x RAD001 monotherapy.

**Figure 2:**
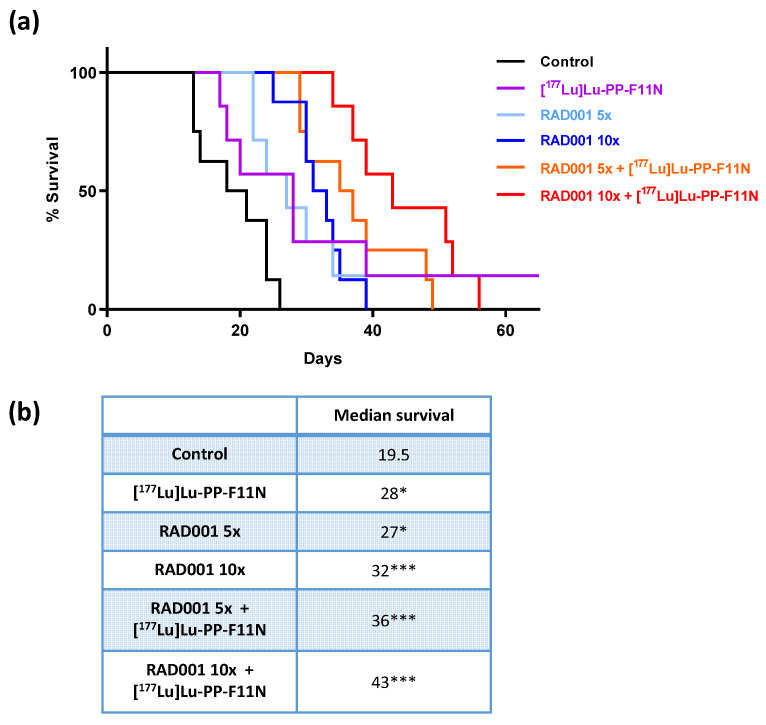
Prolonged survival in RAD001 and [^177^Lu]Lu-PP-F11N-treated mice. (**a**) Survival rates presented as Kaplan–Meier curves of the control and treated mice. (**b**) Significantly extended median survival in treated groups, compared to the control group. * *p* < 0.05, *** *p* < 0.001.

All treatments increased survival compared to the control group (Figure 2a). The median survival in the control group was 19.5 days, whereas the median survival of mice treated with [^177^Lu]Lu-PP-F11N, 5 or 10 doses of RAD001, and 5 or 10 doses of RAD001 in combination with [^177^Lu]Lu-PP-F11N was extended to 28, 27, 32, 36, and 43 days, respectively (Figure 2b).

### 3.2. Histopathology of the Stomach and Kidney

**Figure 3:**
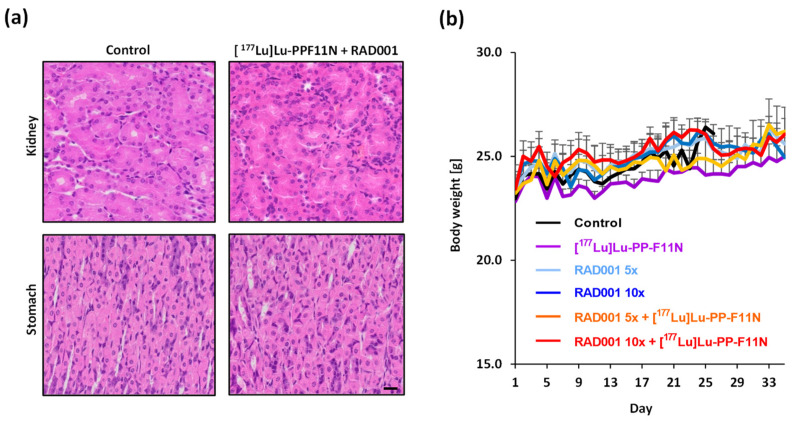
Histological analysis of organs and body weight gain in treated mice. (**a**) Representative images of H&E stains of kidney and stomach of untreated control and [^177^Lu]Lu-PP-F11N-/ RAD001-treated mice, respectively. Scale bar: 20 μm. (**b**) Body weight of A431/CCKBR xenografted nude mice, in the control and all treatment groups. Values show mean ± SD.

To check the potential toxicity of [^177^Lu]Lu-PP-F11N to healthy organs, kidney and stomach from control (PBS), and 10× RAD001 and [^177^Lu]Lu-PP-F11N-treated mice were harvested 13 and 25 days after radiopeptide injection, respectively, and subjected to hematoxylin and eosin (HE) staining. Tissue sections from mice that had received a combinatory treatment did not show any differences compared to the untreated controls (Figure 3a), excluding the acute radiation toxicity to stomach and kidney. During therapy, body weight continuously increased, and no significant differences between control and treated mouse groups were noted (Figure 3b).

## 4. Discussion

The mTORC1 signaling pathway is a major regulator of protein synthesis [11]. Hyperactivation of mTORC1 was reported in many human cancers, including CCKBR-positive primary MTC and lymph node metastases [12], whereas mTORC1 inhibition is known to reduce tumor angiogenesis and induce apoptosis as well as to enhance cancer sensitivity towards therapy-induced DNA damage [13]. Hence, mTORC1 inhibitors were recommended for further development not only as a monotherapy, but also for combination with external radiotherapy. In line with previous findings, we demonstrated improved in vivo therapeutic efficacy in A431/CCKBR-tumor bearing nude mice treated with the mTORC1 inhibitor RAD001 (everolimus) and the radiolabeled minigastrin analogue [^177^Lu]Lu-PP-F11N. The superior effect of combinatory treatment over monotherapy with either RAD001 or [^177^Lu]Lu-PP-F11N alone can be explained by the increased expression of CCKBR receptors due to the TORC1 signaling cascade inhibition, and subsequently increased uptake of radiolabeled minigastrin, as previously reported [8]. In this latter study, the enhanced uptake of the radiolabeled minigastrin was specific to CCKBR-expressing tumors, whereas the uptake in the gastrointestinal tract, which expresses endogenous CCKBR, was unchanged, presumably due to the fact that, in contrast to healthy tissue, cancers have high activity of mTORC1. A higher accumulation of the radioactive peptide leads to significantly increased DNA damage, which correlates with cancer cytotoxicity [14]. Furthermore, allosteric inhibitors of mTORC1, such as rapamycin analogues (rapalogs, including RAD001), exert anti-cancer activity and can sensitize cancer cells to radiotherapy [15,16]. Thus, as indicated by our study, the efficacious combinatory treatment with RAD001 and [^177^Lu]Lu-PP-F11N presumably results from the increased uptake of [^177^Lu]Lu-PP-F11N as well as from the radiosensitizing and anti-cancer effects of mTORC1 inhibition. Similarly to our results, radiotherapy using a [^177^Lu]Lu-labeled gastrin-releasing peptide receptor (GRPR) antagonist [^177^Lu]Lu-RM2 in combination with mTORC1 inhibitor rapamycin significantly prolonged survival without treatment-related toxicity in a preclinical in vivo model, when compared to single treatment with either agent alone [17]. Interestingly, in that latter study, there was a trend for a higher tumor uptake of radiolabeled RM2 in rapamycin-treated nude mice bearing PC-3 tumors. Yet, the molecular mechanism for the increased uptake and radiosensitizing effects of mTORC1 inhibitor rapamycin to [^177^Lu]Lu-RM2 in GRPR-positive cancers has not been elucidated and this point warrants further investigation.

In clinical settings, the pharmacokinetics and safety of RAD001 (everolimus) did not show dose-limiting toxicities in patients with solid tumors, including colorectal, lung, esophageal, and gastric cancer [18]. Furthermore, everolimus showed antitumor activity at a relatively low toxicity in patients with advanced MTC [19], and was previously approved for the treatment of advanced renal cell carcinoma and subependymal giant cell astrocytoma [20,21]. More recently, a phase I study in patients with advanced progressive gastro-entero pancreatic neuroendocrine tumors established maximum tolerated everolimus doses of 7.5 mg daily over 24 weeks in combination with the FDA-approved [^177^Lu]Lu-octreotate (Lutathera^®^), which targets somatostatin receptor 2 [22]. In the latter study, an overall response rate of 44% was observed, and no patient progressed during treatment. In our study, we did not observe any significant side effects in mice treated with [^177^Lu]Lu-PP-F11N and RAD001. However, long-term toxicity cannot be excluded. A recent clinical trial with radiolabeled minigastrin in patients with advanced MTC showed the specific accumulation of [^177^Lu]Lu-PP-F11N in neoplastic tissue and low adverse reactions with favorable biodistribution at low doses of renal and bone marrow irradiation [7]. The study proposed the stomach as a dose-limiting organ and further dosimetry-based estimates of fractionated therapy with a cumulative activity of 50 GBq were considered possible without exceeding the maximum tolerated dose (MTD) for the stomach. Nevertheless, more studies are needed to evaluate the MTD and efficacy of [^177^Lu] Lu-PP-F11N in cancer patients. In summary, together with previously studies, our present data indicate that the combination of everolimus with peptide receptor radionuclide therapy significantly improves therapeutic efficacy without increasing toxicity.

## 5. Conclusions

The present study demonstrates the superior therapeutic efficacy of the combinatory treatment with RAD001 and [^177^Lu]Lu-PP-F11N over monotherapy without severe adverse effects in a preclinical mouse model of CCKBR-positive cancer. Patients with CCKBR-positive tumors can benefit from the combinatory treatment with RAD001 and radioactive minigastrin analogue due to its improved efficacy at low toxicity.

## 6. Patents

Part of the results of this study has been used for the patent application.

## Supplementary Materials

The following are available online at https://www.mdpi.com/article/10.3390/pharmaceutics13122156/s1: Figure S1: Efficiency of PP-F11N Radiolabeling.
