# Supplementary material for: Therapeutic Response of CCKBR-Positive Tumors to Combinatory Treatment with Everolimus and the Radiolabeled Minigastrin Analogue [177Lu]Lu-PP-F11N
Source: Pharmaceutics. 2021 Dec 15;13(12):2156. doi: 10.3390/pharmaceutics13122156 (PMC8708304; doi:10.3390/pharmaceutics13122156)
Supplement: Supplementary file 1 [file pharmaceutics-13-02156-s001.zip › pharmaceutics-1494913-supplementary.pdf]

pharmaceutics

MDPI

Supplementary Materials: Therapeutic Response of CCKBR-Positive Tumors to Combinatory Treatment with Everolimus and the Radiolabeled Minigastrin Analogue [^{177}Lu]Lu-PP-F11N

Michal Grzmil, Stefan Imobersteg, Alain Blanc, Stephan Frank, Roger Schibli and Martin P. Béhé

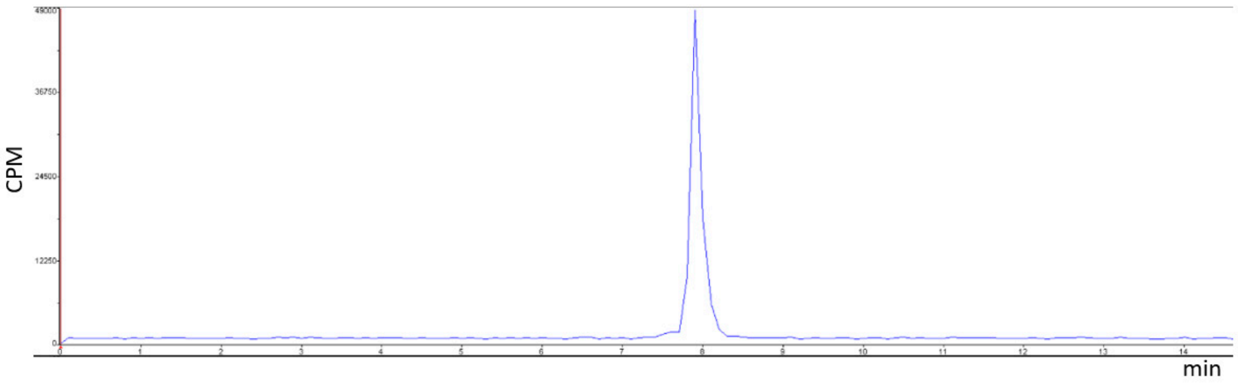

Figure S1. Efficiency analysis of PP-F11N radiolabeling.

The reverse-phase HPLC chromatogram for Lu-177 labeled PP-F11N. The labeling was carried out as described in material and methods and reached above 99 % efficiency. Retention time; 7.9 min. CPM; Counts per minute.
